# Retraction: INPP1 up‐regulation by miR‐27a contributes to the growth, migration and invasion of human cervical cancer

**DOI:** 10.1111/jcmm.17859

**Published:** 2023-07-24

Pu Li, Qiaoge Zhang and Hua Tang. *J Cell Mol Med*. 2019; 23: 7709–7716. The above article, published online on 26 September 2019 on Wiley Online Library (https://onlinelibrary.wiley.com/doi/full/10.1111/jcmm.14644), has been retracted by agreement between the journal Editor‐in‐Chief, Stefan Constantinescu and John Wiley & Sons Ltd.

The retraction has been agreed following an investigation based on allegations raised by a third party, indicating irregularities in figure panels for manuscripts in multiple journals. Irregularities and concerns were found in Figures 2D, 2E, 4C and 4D involving eight images in the article. The authors confirmed the errors. Raw data underlying these figure panels were no longer available and as a result, the journal cannot independently verify the conclusions of the article. The authors were contacted regarding the findings and the proposed retraction, but we did not receive a response.

